# INTEREST GROUP SESSION—RAINBOW RESEARCH GROUP: ADAPTING AN EVIDENCE-BASED INTERVENTION TO LGBT ADULTS WITH DEMENTIA AND CARE PARTNERS: MOBILIZING SUPPORT NETWORKS

**DOI:** 10.1093/geroni/igz038.1246

**Published:** 2019-11-08

**Authors:** Karen Fredriksen Goldsen, Linda Teri

**Affiliations:** University of Washington, Seattle, Washington, United States

## Abstract

LGBT (lesbian, gay, bisexual, and transgender) older adults have been found to have elevated risks of cognitive impairment. Maintaining quality of life is a challenge for those experiencing cognitive decline and their caregivers. Whereas support networks are essential for quality of life, LGBT older adults with dementia may face unique risks, such as stigma, social isolation, lack of family support, and barriers to healthcare. Aging with Pride: IDEA (Innovations in Dementia Empowerment and Action), is the first federally funded clinical trial to test an intervention designed to improve quality of life of LGBT older adults with dementia and caregivers adapting a preexisting program teaching behavioral strategies and physical exercises. The intervention incorporated empirical findings from a longitudinal study, Aging with Pride: National Health, Aging, and Sexuality/Gender Study (NHAS) and developed innovative and culturally responsive approaches. Kim and colleagues examine predictors of longitudinal changes in physical functioning among LGBT older adults with cognitive impairment focusing on physical, social, and recreational activities as well as stigma. Emlet and colleagues investigate caregiving experiences among LGBT older adults and identify factors that are associated with their physical and mental health. Lastly, Fredriksen Goldsen and colleagues introduce how the modifiable factors identified from the Aging with Pride: NHAS were incorporated in the IDEA intervention and evaluate the processes of the culturally-responsive approaches implemented in the study. The presentations in this symposium illustrate the importance of tailoring clinical trial studies for hard-to-reach and underserved populations with dementia responding to their unique health needs.

